# Characterization of a Novel *Lentzea* Species Isolated from the Kumtagh Desert and Genomic Insights into the Secondary Metabolite Potential of the Genus

**DOI:** 10.3390/microorganisms13071628

**Published:** 2025-07-10

**Authors:** Ying Wen, Jiahui Li, Fujun Qiao, Wanyin Luo, Tuo Chen, Guangxiu Liu, Wei Zhang

**Affiliations:** 1 State Key Laboratory of Ecological Safety and Sustainable Development in Arid Lands, Northwest Institute of Eco-Environment and Resources, Chinese Academy of Sciences, Lanzhou 730000, China; wenying19@mails.ucas.ac.cn (Y.W.);; 2 Key Laboratory of Extreme Environmental Microbial Resources and Engineering of Gansu Province, Northwest Institute of Eco-Environment and Resources, Chinese Academy of Sciences, Lanzhou 730000, China; 3 University of Chinese Academy of Sciences, No. 19A Yuquan Road, Beijing 100049, China; 4 School of Biological and Pharmaceutical Engineering, Lanzhou Jiaotong University, Lanzhou 730000, China; 5 State Key Laboratory of Cryospheric Science and Frozen Soil Engineering, Northwest Institute of Eco-Environment and Resources, Chinese Academy of Sciences, Lanzhou 730000, China

**Keywords:** *Lentzea xerophila*, polyphasic taxonomy, Kumtagh desert, secondary metabolite potential

## Abstract

A novel actinobacterial strain, designated E54^T^, was isolated from a hyper-arid desert soil sample collected from the Kumtagh Desert in Dunhuang, Gansu Province, China. Phylogenetic analysis based on 16S rRNA gene sequences placed strain E54^T^ within the genus *Lentzea*, showing highest similarity to *Lentzea waywayandensis* DSM 44232^T^ (98.9%) and *Lentzea flava* NBRC 15743^T^ (98.5%). However, whole-genome comparisons revealed that the average nucleotide identity (ANI) and digital DNA–DNA hybridization (dDDH) values between E54^T^ and these related strains were below the thresholds for species delineation. Strain E54^T^ exhibited typical morphological characteristics of the genus *Lentzea*, forming a branched substrate. It grew optimally at 28–30 °C, pH 7.0–9.0, and tolerated up to 10% NaCl. The cell wall contained meso-diaminopimelic acid, the predominant menaquinone was MK-9(H_4_), and major fatty acids included iso-C_16:0_. The polar lipid profile comprised diphosphatidyl glycerol, phosphatidyl ethanolamine, phosphatidyl inositol, hydroxyphosphatidyl ethanolamine, and an unidentified lipid. The characteristic amino acid type of the cell wall was meso-DAP. Whole-cell hydrolysis experiments revealed the characteristic cell wall sugar fractions: ribose and galactose. The genome of strain E54^T^ is approximately 8.0 Mb with a DNA G+C content of 69.38 mol%. Genome mining revealed 39 biosynthetic gene clusters (BGCs), including non-ribosomal peptide synthetases (NRPS), polyketide synthases (PKS), terpenes, and siderophores. Comparative antiSMASH-based genome analysis across 38 *Lentzea* strains further demonstrated the genus’ remarkable biosynthetic diversity. NRPS and type I PKS (T1PKS) were the most prevalent BGC types, indicating a capacity to synthesize structurally complex and pharmacologically relevant metabolites. Together, these findings underscore the untapped biosynthetic potential of the genus *Lentzea* and support the proposal of strain E54^T^ as a novel species. The strain E54^T^ (=JCM 34936^T^ = GDMCC 4.216^T^) should represent a novel species, for which the name *Lentzea xerophila* sp. nov. is proposed.

## 1. Introduction

Soil microorganisms possess remarkable biosynthetic capabilities [1,2] and have evolved diverse ecological adaptation strategies to survive under multiple environmental stresses. The unique physiological adaptation mechanisms of soil microorganisms make them an important group of organisms in deserts, playing a significant role in the geochemical cycles of elements such as carbon and nitrogen [3,4,5]. Actinomycetes are core members of the primary microbial community in soil. They not only participate in the decomposition of organic matter and complex polymers in soil, promoting global carbon cycling, but also enhance plant productivity and stress resistance [6,7]. In addition, actinomycetes are best known for their ability to produce large quantities of biologically active compounds with important applications [8,9,10]. Recent studies have revealed that in extreme environments such as polar and arid deserts, the abundance of biosynthetic gene clusters (BGCs), particularly those encoding non-ribosomal peptide synthetases (NRPSs) and polyketide synthases (PKS) [11], is often negatively correlated with environmental factors such as moisture, carbon, and nitrogen availability [12]. Furthermore, several bioactive natural products have been isolated from desert-derived actinobacteria, including antifungal compounds from *Saccharothrix* SA198 in the Sahara Desert [13], abenquines from *Streptomyces* DB634 in the Atacama Desert [14], and multiple translation-inhibiting compounds from strains isolated from the Taklamakan Desert, such as adamycin derivatives and acetylated griseoviridin analogs [15,16]. These findings highlight the untapped biosynthetic potential of desert-dwelling microbes and the urgent need to explore them for novel bioactive molecules.

The genus *Lentzea*, belonging to the family Pseudonocardiaceae within the order Actinomycetales, comprises Gram-positive, aerobic, and non-motile actinobacteria with well-developed branched mycelia and rod-shaped elements. Initially proposed by Yassin et al. in 1995 [17], it was reclassified by Labeda et al. in 2001 [18] based on chemotaxonomic and phylogenetic data. Members of *Lentzea* have been isolated from diverse ecological habitats including desert soils [19,20], sediments of the Coal Basin [21] and Tibetan Plateau [22], Guizhou karst limestone [23], and so on. These bacteria are often oligotrophic and are presumed to contribute to organic matter decomposition and biogeochemical cycling [24]. Owing to their ecological resilience and metabolic potential, *Lentzea* species have been reported to produce several noteworthy bioactive compounds, including the following: (i) lentzeosides with anti-HIV-1 integrase activity from *Lentzea* sp. H45 [25]; (ii) antimicrobial [22,25]; (iii) other biological functional activities [26,27,28].

Despite these promising discoveries, systematic studies of *Lentzea* species derived from desert environments remain scarce. Their adaptive evolution under extreme arid conditions and associated metabolic diversity remain poorly understood. In this study, we report the isolation and polyphasic taxonomic characterization of a novel *Lentzea* species, strain E54^T^, from the Kumtagh Desert in northwestern China. Using whole-genome sequencing and phylogenomic analysis, we elucidate the taxonomic status of E54^T^ and place it within a genus-wide, high-resolution phylogenetic framework constructed from all publicly available *Lentzea* genomes. Furthermore, we perform comparative genomic and biosynthetic potential analyzes to assess secondary metabolite diversity across the genus. Our findings expand the current understanding of *Lentzea* species in extreme environments and provide valuable genomic resources for future drug discovery and ecological studies.

## 2. Materials and Methods

### 2.1. Isolation and Culture Maintenance

Strain E54^T^ was isolated in August 2022 from a surface soil sample collected in the hyper-arid Kumtagh Desert near Dunhuang City, Gansu Province, Northwest China (39.6656° N, 94.3773° E; elevation 1596.6 m). Approximately 1 g of soil was suspended in sterile distilled water, shaken vigorously for 5 min, and serially diluted up to 10^−5^. Aliquots of each dilution were spread onto R_2_A agar plates and incubated aerobically at 25 °C for 15 days. Distinct colonies were repeatedly streaked on fresh R_2_A plates to obtain a pure culture. The purified isolate was preserved in 20% (*v*/*v*) glycerol at −80 °C for long-term storage. For phylogenetic and phenotypic comparisons, two closely related type strains—*Lentzea flaviverrucosa* CGMCC 4.578^T^ and *Lentzea *albidocapillata** subsp. *violacea* CGMCC 4.2093^T^—were selected based on 16S rRNA gene sequence similarity and obtained from China General Microbiological Culture Collection Center (CGMCC).

Morphological characteristics of strain E54^T^ and the reference strains were examined after growth on a variety of media, including ISP media [29], nutrient agar, Bennett’s agar [30], and Czapek’s Dox agar [31]. Following 2 weeks incubation at 30 °C, all culture media refer to the composition provided by DSMZ (https://www.bacmedia.dsmz.de/medium, accessed on 6 July 2023). Strain was observed by scanning electron microscopy (SEM) by first growing the strain on ISP2 for 28 °C, pH 7.0 for 14 days.

### 2.2. Phylogenetic Analysis Based on 16S rRNA Gene Sequences

Genomic DNA extraction was carried out according to previously established protocols [32,33]. The full-length 16S rRNA gene sequence was retrieved from the annotated genome. Sequence similarity searches conducted using the EzBioCloud database [34] revealed that strain E54^T^ exhibited the highest sequence similarity to *Lentzea flaviverrucosa* (98.56%) and *Lentzea albidocapillata* subsp. *violacea* (98.34%), indicating its classification within the genus *Lentzea*. Phylogenetic trees were constructed using the maximum-likelihood method in MEGA7 software (https://www.megasoftware.net/older_versions/ 7.0 (CC for 64-bit Windows)) [35]. Sequence alignments were performed with ClustalW [36], followed by manual refinement to ensure consistent alignment. A distance matrix was calculated using the p-distance method [37], and the robustness of the tree was assessed with bootstrap analysis using 1000 replicates [38].

### 2.3. Genome Sequencing and Annotation

Strain E54 cells were collected after shaking in R_2_A medium at 30 °C for 2 days, with centrifugation to remove the supernatant. Genomic DNA extraction was performed using the OMEGA Bacterial DNA Kit (Norcross, GA, USA, D3350-02), in accordance with the manufacturer’s guidelines. Whole-genome sequencing for strain E54^T^ was performed on the Pacific Biosciences Sequel II platform. De novo assembly of the genome was carried out using the HGAP4 pipeline integrated in SMRT Link v8.0 [39]. Genome annotation was performed with Prokka v1.13.4 [40], and secondary metabolite biosynthetic gene clusters (BGCs) were identified using antiSMASH v7.0 under the default parameters [41]. For statistical analysis, repeated calculations were performed on composite secondary metabolite gene cluster. To compare the genomes, average nucleotide identity (ANI) values were calculated with FastANI v1.34 [42], and digital DNA–DNA hybridization (dDDH) values were estimated using the Genome-to-Genome Distance Calculator (GGDC) v3.0 (https://ggdc.dsmz.de/ggdc.php, accessed on 8 May 2024) [43]. The assembled genome of strain E54^T^ has been deposited in the NCBI GenBank under the BioProject accession number PRJNA1272222.

To further clarify the phylogenetic and evolutionary relationships, genomes of 37 strains showing the highest 16S rRNA gene similarity to strain E54^T^ were retrieved from public databases. For strains without available genome sequences, lyophilized cultures were obtained from culture collections and subjected to whole-genome sequencing as described above.

### 2.4. Physiological and Chemotaxonomic Characterization

#### 2.4.1. Cultural and Phenotypic Characteristics

Strain E54^T^ was cultured on various standard media, including ISP 2–7, nutrient agar, Czapek’s agar, and Bennett’s agar, and was incubated at 28 °C for 14 days. Morphological features such as colony color, aerial and substrate mycelium pigmentation, diffusible pigments, and melanin production were recorded. Growth temperature range was tested on R_2_A agar at intervals of 5 °C from 5 to 50 °C. The pH growth range (4.0–13.0, interval 1.0) was evaluated in R_2_A broth adjusted with appropriate buffer systems (KH_2_PO_4_/HCl, KH_2_PO_4_/K_2_HPO_4_, K_2_HPO_4_/NaOH). Salt tolerance was determined using R_2_A agar supplemented with 1–15% (*w*/*v*) NaCl at 1% intervals. Physiological and biochemical features including utilization of single-carbon and -nitrogen sources, gelatin liquefaction, starch hydrolysis, and urea degradation were assessed using established protocols. Cultural characteristics are provided in Table S1.

#### 2.4.2. Chemotaxonomic Analyses

Biomass for chemotaxonomic analysis was obtained by growing strain E54^T^ in ISP 2 broth at 28 °C, 300 rpm for 4 days. Cells were harvested by centrifugation, washed with distilled water, and freeze-dried. The diaminopimelic acid (DAP) in the cell wall was determined using the method of Staneck and Roberts [44]. Whole-cell sugars and DAP were examined by thin-layer chromatography (TLC) using established protocols. Cellular fatty acid methyl esters (FAMEs) were extracted according to Sasser’s method [45] and subsequently analyzed with the Sherlock Microbial Identification System (MIDI) [46]. Menaquinones were isolated from freeze-dried cells, purified as outlined by Collins [47], and analyzed via HPLC with UV detection [48]. Polar lipids were extracted and identified using two-dimensional TLC, following the procedure described by Minnikin et al. [49].

## 3. Results

### 3.1. Morphological, Physiological, and Chemotaxonomic Characteristics of Strain E54^T^

**Figure 1:**
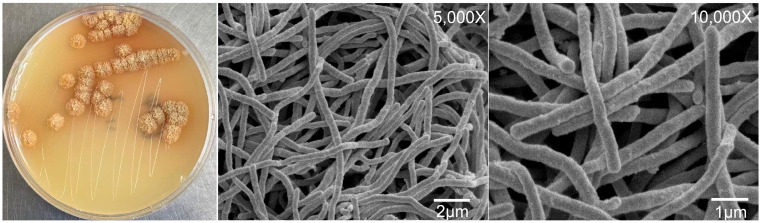
Colony and scanning electron micrograph of strain E54^T^ growing on ISP2 at 28 °C for 14 days.

After 14 days of cultivation on ISP2 medium at 28 °C, strain E54^T^ formed compact, circular colonies with well-developed substrate mycelia. The aerial mycelia were irregularly branched, wrinkled, and light brown in color, but no spore formation was observed. Scanning electron microscopy revealed abundant aerial hyphae, which were rod-like, smooth-surfaced, and moderately flexuous (Figure 1).

Strain E54^T^ exhibited good growth on nutrient agar, ISP1, ISP2, ISP3, ISP5, and Czapek’s agar, while weaker growth was observed on ISP4, ISP6, and ISP7. No sporulation was detected on any of the tested media. Both substrate and aerial mycelia were formed on most media. Soluble pigments were yellow on ISP2, ISP3, ISP4, ISP5, and ISP6; brownish yellow on ISP7 and Bennett’s agar; and absent (white substrate mycelia) on nutrient agar and Czapek’s agar (Table S1). The optimal growth conditions for strain E54^T^ were 30 °C and pH 7.0, with growth occurring in the ranges of 20–40 °C and pH 6.0–10.0. It tolerated NaCl concentrations of 0–5%, with optimal growth at 0–2%. In comparison, *L. flaviverrucosa* CGMCC 4.578^T^ failed to grow above 3% NaCl, and *L. albidocapillata* subsp. *violacea* CGMCC 4.2093^T^ tolerated up to 4% NaCl.

A comparison of phenotypic traits among strain E54^T^ and its closest relatives, *L. flaviverrucosa* CGMCC 4.578^T^ and *L. albidocapillata* subsp. *violacea* CGMCC 4.2093^T^, revealed several distinguishing features (Table 1). While the three strains showed similar utilization profiles for arabinose, lactose, maltose, and fructose, strain E54^T^ demonstrated a notably higher capacity for galactose utilization. In contrast, the reference strains showed greater ability to metabolize raffinose, mannitol, sucrose, inositol, and sorbitol.

Strain E54^T^ could utilize several amino acids—including L-histidine, L-proline, L-alanine, L-serine, L-phenylalanine, and L-asparagine—as sole nitrogen sources, but could not grow with L-glutamate alone. It showed robust growth on media containing alanine, glycine, lysine, tyrosine, proline, serine, aspartate, or histidine as sole nitrogen sources. Notably, it exhibited stronger growth on tyrosine-containing media compared to the two reference strains.

Chemotaxonomic analysis revealed that the peptidoglycan of strain E54^T^ contained meso-diaminopimelic acid (meso-DAP), which is a characteristic feature of the *Lentzea* genus. The diagnostic whole-cell sugars identified were ribose and galactose. The predominant menaquinone detected was MK-9(H_4_). Major polar lipids included diphosphatidyl glycerol (DPG), phosphatidyl ethanolamine (PE), phosphatidyl inositol (PI), and hydroxyphosphatidyl ethanolamine (OH-PE). The major fatty acid was iso-C_16:0_ (33.97%). For comparison, *L. flaviverrucosa* CGMCC 4.578^T^ primarily contained summed feature 7 (32.3%) and iso-C_16:0_ (20.98%), whereas *L. albidocapillata* subsp. *violacea* CGMCC 4.2093T predominantly contained iso-C_16:0_ (32.5%). The characteristic amino acid type of the cell wall was meso-DAP. Whole-cell hydrolysis experiments revealed the characteristic cell wall sugar fractions: ribose and galactose.

### 3.2. Phylogenetic Analysis

**Figure 2:**
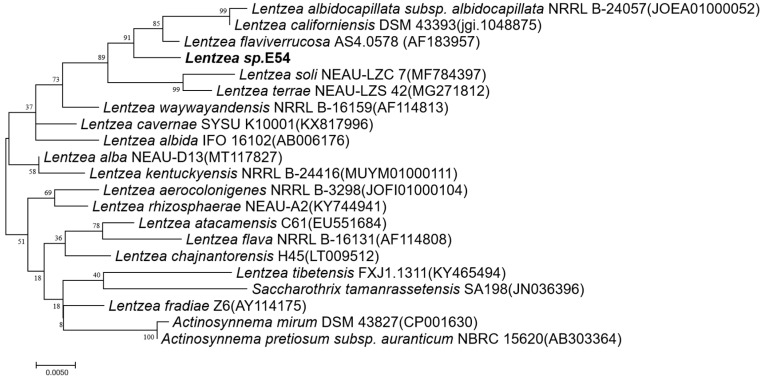
Phylogenetic tree of strain *Lentzea* sp. E54 and its similar strains based on 16S rRNA genes (maximum-likelihood method).

The analysis of the 16S rRNA gene sequence showed that strain E54^T^ shared the greatest similarity with *Lentzea flaviverrucosa* AS4.0578^T^ (98.56%) and *Lentzea albidocapillata* subsp. *violacea* IMSNU 50388T (98.34%). Phylogenetic analysis using 16S rRNA gene sequences revealed that strain E54T clustered with *Lentzea flaviverrucosa* AS4.0578^T^ in the maximum-likelihood tree (Figure 2), indicating its novel taxonomic position within the *Lentzea* genus.

The complete genome of *Lentzea* sp. E54^T^ was sequenced and annotated to elucidate its genetic composition and taxonomic position. The genome of strain E54^T^ is composed of 1 contig with a total length of 9,043,573 bp and a GC content of 69.38%. A total of 8602 coding DNA sequences (CDS) were identified. Notably, strain E54^T^ harbored one of the highest rRNA and tRNA gene counts among all analyzed *Lentzea* strains, suggesting potential for enhanced translational capacity and adaptive versatility.

To place strain E54T in context, genome features of 37 publicly available *Lentzea* genomes were examined. Genome sizes among the *Lentzea* species ranged from 7.7 Mb to 10.8 Mb, with CDS numbers varying from 7789 to 12,448, and tRNA counts spanning 2 to 15 copies. The largest genome was observed in *Lentzea aerocolonigenes* NRRLB-16138 (12,448 CDS across 10,637,170 bp), while the smallest belonged to *Lentzea* sp. CMU235 (8154 CDS; 7.7 Mb). A summary of genome assembly metrics across all strains is provided in Supplementary Table S2.

**Figure 3:**
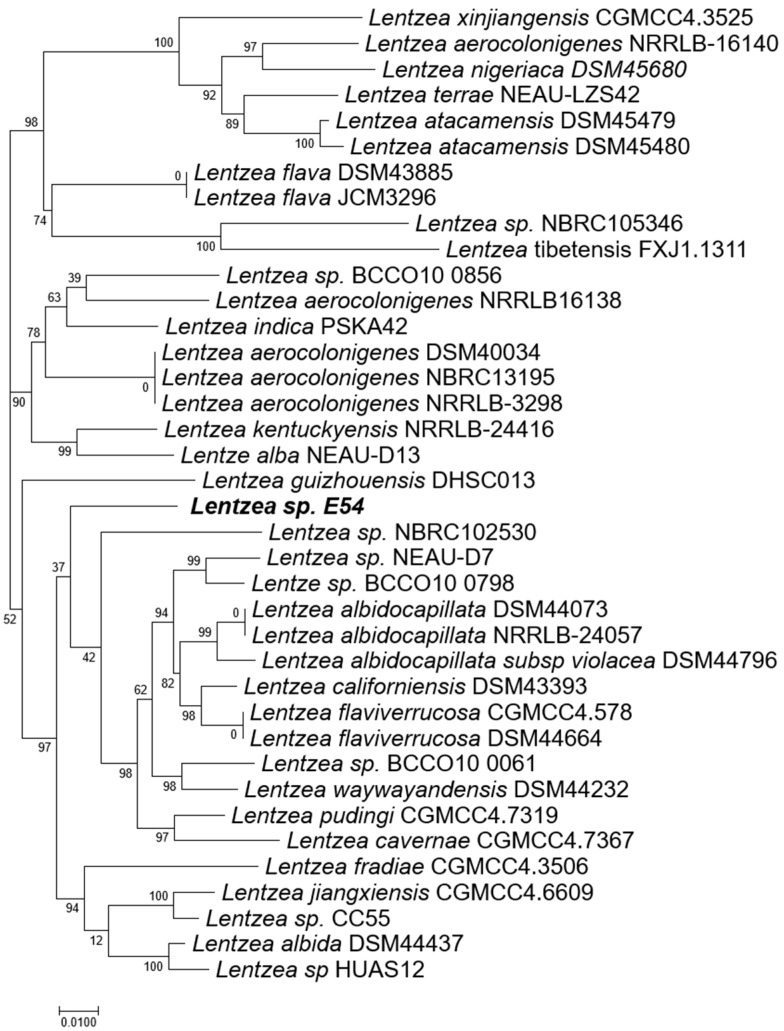
Phylogenetic tree of UBCG constructed on the basis of whole-genome sequences of strain E54^T^ and other closely related conspecifics. The numbers on the branch nodes represent the gene support index (the maximum gene value in the UBCG method is 92).

To further clarify the phylogenetic position of strain E54^T^, we retrieved genome sequences of 37 closely related strains with publicly available data from the 50 top BLAST (https://blast.ncbi.nlm.nih.gov/Blast.cgi?PROGRAM=blastn&PAGE_TYPE=BlastSearch&LINK_LOC=blasthome (accessed on 8 March 2024)) hits based on 16S rRNA similarity. A whole-genome phylogenetic tree was reconstructed using the UBCG pipeline, and pairwise average nucleotide identity (ANI) values were calculated. Consistent with the 16S rRNA tree, strain E54^T^ formed a separate clade in the genome-based phylogeny (Figure 3).

The ANI values between strain E54 and its closest type strains, *L. flaviverrucosa* AS4.0578^T^ and *L. albidocapillata* subsp. *violacea* IMSNU 50388^T^, were 89.86% and 90.09%, respectively (Table 2), which are well below the commonly accepted species delineation threshold of 95–96%. Digital DNA–DNA hybridization (dDDH) values were 37.2% and 37.7%, also lower than the 70% threshold for species demarcation. These results collectively support that strain E54 represents a novel species within the genus *Lentzea*.

### 3.3. Assessment of the Secondary Metabolic Potential of the Genus Lentzea

**Figure 4:**
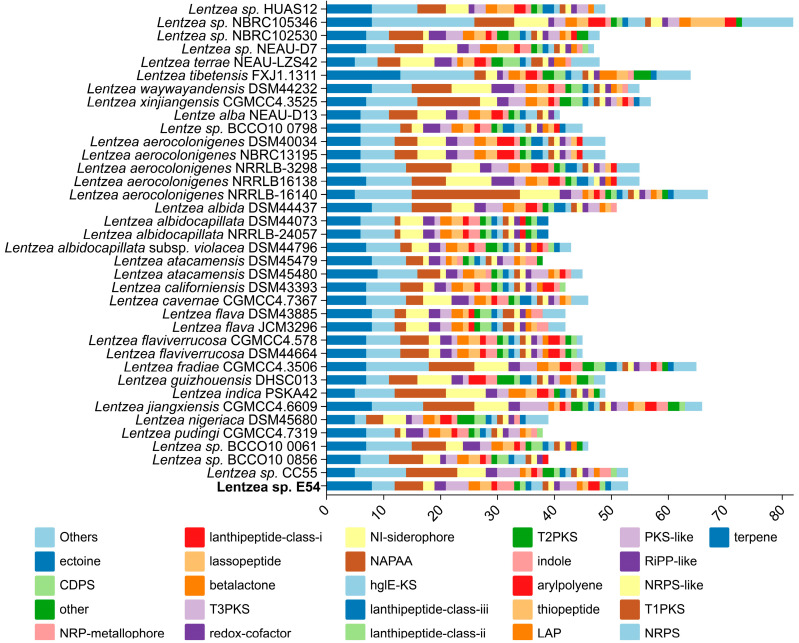
Statistical overview of the biosynthetic gene clusters (BGCs) identified across 38 *Lentzea* strains.

The antiSMASH-based genomic analysis of 38 *Lentzea* strains revealed a broad diversity of biosynthetic gene clusters (BGCs), indicating the genus’ considerable potential for producing bioactive secondary metabolites (Figure 4; the order of the legends is from right to left, with “others” being the last one). The most common BGC types identified were NRPS (non-ribosomal peptide synthetase) and T1PKS (type I polyketide synthase), with *Lentzea* sp. NBRC105346 having the highest number of clusters (18 NRPS and 7 T1PKS), suggesting a strong capacity for synthesizing complex peptides and polyketides. Terpenes were consistently present (5–13 clusters per genome), while NRPS-like, PKS-like, and transAT-PKS clusters further indicated diverse biosynthetic capabilities. Notably, *Lentzea aerocolonigenes* NRRLB-16140 contained a high number of T1PKS clusters (19), and *Lentzea tibetensis* FXJ1.1311 harbored multiple transAT-PKS clusters, both of which are associated with the production of structurally complex and potentially pharmacologically relevant metabolites. In addition, rare and specialized BGCs were detected in specific strains, including lanthipeptides (e.g., *Lentzea* sp. CMU181), betalactones, phosphonates, and thiopeptides, which could be indicative of niche adaptations and unexplored antibiotic potential. The presence of siderophores, ectoine, and redox cofactor clusters further suggests ecological adaptations to stress conditions.

While these results are promising, the bioactive potential of these BGCs requires experimental validation, including heterologous expression and metabolomic profiling, to fully assess their pharmacological and biotechnological applications. Overall, *Lentzea* exhibits a rich secondary metabolome with strain-specific specializations, positioning it as a promising source for future drug discovery and biotechnological applications.

## 4. Conclusions

A novel actinobacterial strain, designated E54^T^, was isolated from hyper-arid desert soil collected from the Kumtagh Desert in northwestern China. Polyphasic taxonomic analysis, including phylogenomic inference, ANI, dDDH, phenotypic characterization, and chemotaxonomic profiling, clearly demonstrated that strain E54^T^ represents a novel species within the genus *Lentzea*. Strain E54^T^ displays several phenotypic features adapted to arid environments, including high salt and pH tolerance. It also exhibits a unique polar lipid profile and produces multiple secondary metabolites with low similarity to known gene clusters. On the basis of these data, strain E54^T^ is proposed as the type strain of a novel species, for which the name *Lentzea xerophila* sp. nov. is proposed.

## 5. Description of *Lentzea xerophila* sp. nov.

*Lentzea xerophila* (Etymology: xe.ro’phi.la. Gr. masc. adj. xêros, dry; N.L. masc. adj. suff. -philus, loving; N.L. fem. adj. xerophila, dry-loving, occurring in dry, xeric habitat. Kumtagh desert in Northwest China, the source of the type strain).

Gram-stain-positive, aerobic, and non-motile actinobacterium that forms extensively branched substrate and aerial mycelia. Aerial mycelium forms long chains of rod-shaped or short cylindrical structures with a wrinkled surface. Growth occurs on ISP 2–ISP 7 and Bennett’s agar. Good growth is observed at 28–37 °C (optimum, 28–30 °C), pH 6.0–10.0 (optimum, 7.0–9.0), and in the presence of up to 10% (*w*/*v*) NaCl. The strain can hydrolyze starch, casein, and cellulose. It produces acid from D-glucose, D-fructose, sucrose, raffinose, and maltose. Nitrate is not reduced. The predominant menaquinone is MK-9(H_4_). The major fatty acid was iso-C_16:0_. The polar lipid profile includes diphosphatidyl glycerol, phosphatidyl ethanolamine, phosphatidyl inositol (PI), hydroxyphosphatidyl ethanolamine, and an unidentified lipid. The characteristic amino acid type of the cell wall was meso-DAP. Whole-cell hydrolysis experiments revealed the characteristic cell wall sugar fractions: ribose and galactose. The DNA G+C content is 69.38 mol%. The genome size is approximately 8.0 Mb and harbors biosynthetic gene clusters encoding non-ribosomal peptides, polyketides, terpenes, and lanthipeptides, most of which show low similarity to known clusters, suggesting untapped secondary metabolic potential.

The type strain E54^T^ (=JCM 37543^T^ = GDMCC 4.414^T^) was isolated from surface soil collected in August 2023 from the hyper-arid Kumtagh Desert, China. Based on whole-genome phylogenomic and chemotaxonomic analyses, the strain belongs to the genus *Lentzea* but represents a novel species. GenBank/EMBL/DDBJ accession number for the genome and 16S rRNA gene sequence of strain E54^T^ are PRJNA1272222 and PP694170.1, respectively.

## Figures and Tables

**Table 1:** Phenotypic properties of strain E54 and related type species.

Characteristic	1	2	3
0~5% (*w*/*v*) NaCl	++	+	–
40 °C	++	–	±
Carbon utilization (1.0%, *w*/*v*):			
D-Galactose	+++	+	++
D-Mannitol	+	++	+++
Inositol	+	+++	++++
Raffinose	++	++++	+++
D-Xylose	+	+	–
Sorbitol	+	++	++++
L-rhamnose	+	++	++
Sucrose	+	+++	++++
Nitrogen utilization (1.0%, *w*/*v*):			
Alanine	+++	+++	+++
Glycine	++	+++	++
Glutamic acid	–	+	+
Lysine	+	+++	++
Tyrosine	++++	+	+++
Proline	++	+++	++++
Serine	+++	+	+++
Aspartic acid	+	+++	++++
Histidine	+	+++	++

Strains: 1, E54; 2, *Lentzea flaviverrucosa* CGMCC4.578^T^; 3, *Lentzea albidocapillata* subsp. *violacea* CGMCC 4.2093^T^. Symbols: ++++, excellent growth; +++, good growth; ++: moderate growth; +, growth possible; ±, possible growth; –, not utilized. All strains can utilize arabinose, D-Lactose, maltose, and D-Fructose, with little difference in growth status.

**Table 2:** Genome comparisons of *strain* E54^T^ and its closely related species.

Strain	Related Species	ANI	DDH
E54^T^	*Lentzea flaviverrucosa*	89.86	37.2
	*Lentzea albidocapillata* subsp. *violacea*	90.09	37.7
	*Lentzea waywayandensis*	90.03	37.9
	*Lentzea californiensis*	89.92	37.4

## Data Availability

The GenBank/EMBL/DDBJ accession number for the 16S rRNA gene sequence and genome of strain E54T are PP694170.1 and PRJNA1272222, respectively. The data can be accessed at https://www.ncbi.nlm.nih.gov/.

## Supplementary Materials

The following supporting information can be downloaded at: https://www.mdpi.com/article/10.3390/microorganisms13071628/s1. Table S1: Cultural characteristics of strain E54 and the type strains of the most closely related species; Table S2: Genomic characteristics of *Lentzea* species used for comparison. Figure S1. GO annotation results of the *Lentzea* sp. E54^T^ genome; Figure S2. KEGG annotation results of the *Lentzea* sp. E54^T^ genome.

## Abbreviations

The following abbreviations are used in this manuscript:

ANI	Average nucleotide identity
dDDH	Digital DNA–DNA hybridization
ISP	International *Streptomyces* Project
ML	Maximum-likelihood
