# Supplementary material for: Characterization of a Novel Lentzea Species Isolated from the Kumtagh Desert and Genomic Insights into the Secondary Metabolite Potential of the Genus
Source: Microorganisms. 2025 Jul 10;13(7):1628. doi: 10.3390/microorganisms13071628 (PMC12300779; doi:10.3390/microorganisms13071628)
Supplement: Supplementary file 1 [file microorganisms-13-01628-s001.zip › microorganisms-3719212-suppl.pdf]

Supplementary figure:

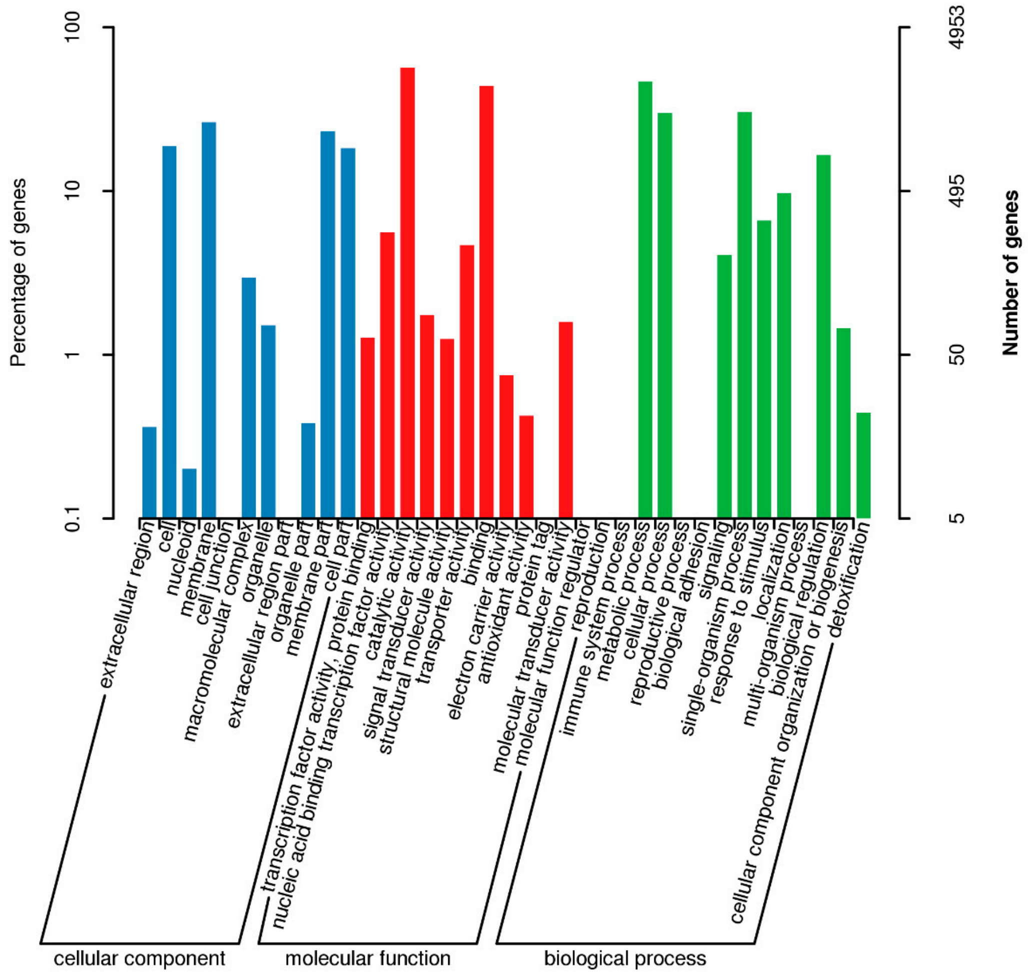

Figure S1. GO annotation results of the *Lentzea* sp. E54^{T} genome.

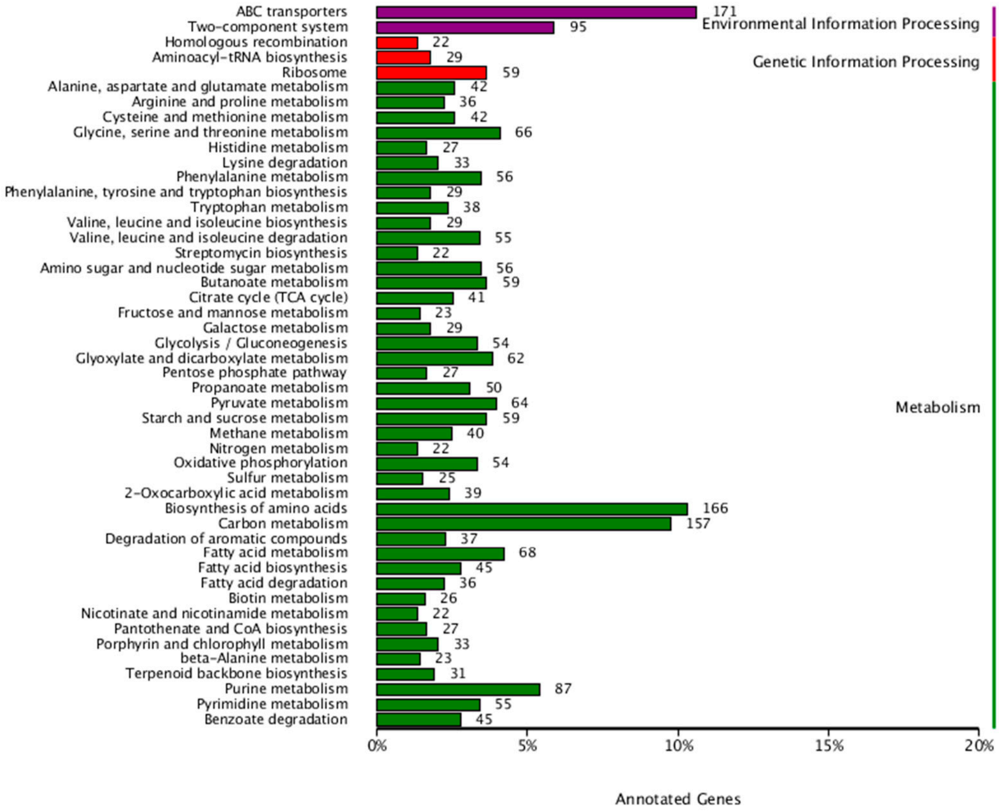

Figure S2. KEGG annotation results of the *Lentzea* sp. E54^{T} genome.
